# Comparison of Tell-Show-Do, Tell-Play-Do and app-based modelling techniques in pediatric dental behaviour management

**DOI:** 10.6026/973206300222975

**Published:** 2026-05-31

**Authors:** Dhwani Dilipbhai Thakkar, Raju Chepyala, Eeraveni Ranadheer, Jurmi Kothari, Shenoy Shailesh Ramdas, Kalisipudi Sandeep, Pratik Surana

**Affiliations:** 1 Department of Dentistry, Doctor Dental, Connecticut, USA; 2 Department of Dental Surgery, Government Medical College & General Hospital, Karimnagar, Telangana, India; 3 Department of Pedodontics and Preventive Dentistry, Government Dental College and Hospital, Ahmedabad, Gujarat, India; 4 Department of Pediatric and Preventive Dentistry, Yenepoya Dental College, Yenepoya University, Mangalore, Karnataka, India; 5 Department of Pediatric and Preventive Dentistry, Lenora Institute of Dental Sciences, Rajahmundry, Andhra Pradesh, India; 6 Department of Pedodontics and Preventive Dentistry, Maitri College of Dentistry and Research Centre, Durg, Chhattisgarh, India

**Keywords:** Dental anxiety, RMS pictorial scale, smart phone app

## Abstract

Managing dental anxiety in children remains a major challenge in pediatric dentistry, as anxious children often demonstrate poor cooperation during dental procedures. Therefore, it is of interest to evaluate and compare the effectiveness of Tell-Show-Do (TSD), Tell-Play-Do (TPD), and smartphone app-assisted modelling techniques in reducing anxiety among pediatric patients. Anxiety was assessed pre- and post-intervention using the RMS Pictorial Scale. All groups showed significant reduction in anxiety scores, with the smartphone app-assisted modelling group demonstrating the greatest improvement. Thus, we show that interactive and technology-assisted behaviour management approaches may offer superior anxiety reduction compared to conventional techniques.

## Background:

Dental anxiety is a common emotional response characterized by feelings of fear, apprehension, or uneasiness toward dental treatment, often without a clearly identifiable cause [1]. In pediatric patients, this anxiety frequently manifests as uncooperative or disruptive behaviour, posing a significant challenge for the dental practitioner [2]. Various stimuli within the dental environment contribute to this fear, including the sound, sight and sensation associated with rotary instruments such as the airotor, as well as procedures like local anaesthetic administration [3]. Effective behaviour management is essential in pediatric dentistry to help children develop positive coping mechanisms and accept dental care without distress [4]. The goal is to reduce fear perception and create a supportive environment that encourages cooperation [5]. A range of non-pharmacological and pharmacological techniques are recommended for managing child behaviour, including TSD, reinforcement, modelling, voice control and contemporary methods such as sedation and general anaesthesia when required [6, 7]. Among these, TSD is widely utilized in clinical practice and is based on gradual familiarization, wherein the child is informed, shown and then exposed to the procedure in a controlled manner [7]. However, considering that children have limited cognitive ability to understand abstract explanations, more interactive approaches have been introduced [8]. The TPD technique incorporates play-based learning, allowing children to handle and explore dental instruments in a non-threatening way, thereby improving understanding and reducing anxiety [9]. With the increasing use of digital technology, smartphone-based applications have emerged as innovative tools for behavior management. These applications provide audiovisual demonstrations of dental procedures in an engaging and child-friendly format, helping to familiarize children with the clinical environment before treatment. Despite the growing popularity of such approaches, limited literature is available comparing these modern techniques with conventional methods [10]. Therefore, it is of interest to report TSD, TPD and smartphone app-assisted modelling techniques for managing anxiety among pediatric dental patients.

## Materials and Methods:

The investigation was designed as a randomized clinical trial and included 30 children aged 6-8 years requiring restoration of a mandibular molar in the Department of Pediatric and Preventive Dentistry. The sample consisted of children showing Frankl's behaviour rating of 3 or 4, indicating positive or definitely positive behaviour, ensuring that participants were cooperative and suitable for non-pharmacological behaviour management techniques. Only children diagnosed with dental caries showing enamel lesions without evidence of dentin involvement (ICDAS score 3) were included. These cases were specifically selected as they could be managed without the administration of local anaesthesia, thereby minimizing additional anxiety factors such as needle fear. Children with systemic illnesses, special healthcare needs, a history of previous negative dental experiences, or those requiring emergency dental care were excluded from the study to maintain uniformity in the sample. After obtaining informed consent from parents or guardians, participants were categorized into three groups (n = 10 each) using a computer-generated random sequence.

## Group I (TSD):

Individuals in this group underwent with the standard TSD technique. The procedure was first explained in simple, child-friendly language (Tell), followed by demonstration using dental instruments without performing the actual procedure (Show). Finally, the restorative procedure was carried out (Do).

## Group II (TPD):

Within this group, children were introduced to dental procedures through a play-based approach. Toy-based dental instruments (Dentist Kit, ZIWKI Smart Toys Co., Ltd., China) were used to simulate dental procedures. Children were encouraged to handle and play with these instruments, mimicking the dental procedure in a non-threatening environment before the actual treatment was performed.

## Group III (Smartphone app-Assisted modelling):

A smartphone-based dental application was provided to the children ("Little Lovely Dentist," Leaf Cottage Games, USA) prior to treatment. The application allowed children to visualize and interact with simulated dental procedures, thereby familiarizing them with the clinical steps and reducing fear through audiovisual modelling. All restorative procedures were performed under standardized clinical conditions by the same operator to eliminate operator variability. Anxiety among children was assessed using the RMS Pictorial Scale (RMS-PS), a validated instrument for pediatric dental anxiety. The scores were recorded at two time points: before the intervention (pre-operative) and completion of the restorative procedure (post-operative). Collected data were compiled and performed by SPSS software (version 25.0; IBM Corp., USA). Within-group changes were analyzed using the paired t-test, while between-group differences were assessed using one-way ANOVA with post-hoc tests.

## Results:

Thirty children within the age group of 6-8 years were enrolled and distributed equally across three groups. The demographic variables, including age and gender distribution, were comparable among all groups, with showing no statistical difference (p > 0.05) (Table 1). Analysis within groups revealed a significant reduction in RMS-PS scores from pre-operative to post-operative measurements in all three groups (p < 0.05), indicating decreased anxiety across all techniques (Table 2). Post-hoc intergroup analysis demonstrated significant differences among the groups (p < 0.05). Anxiety reduction was highest in the smartphone app-assisted modelling group, followed by the Tell-Play-Do group and lowest in the Tell-Show-Do group. Pairwise comparisons further confirmed significant differences across all groups (Table 3).

## Discussion:

Behaviour management is a fundamental aspect of pediatric dental practice, as a child's cooperation directly influences the quality and success of treatment. A child's initial dental appointment is vital in influencing their perception of future dental treatment. Effective behaviour management techniques aim to reduce fear and anxiety, improve communication and establish a trusting relationship between the dentist, child and parent, ultimately promoting a positive dental experience [11, 12]. A variety of behaviour management techniques are available, ranging from basic non-pharmacological approaches to advanced pharmacological methods [13]. Among these, Tell-Show-Do and modelling techniques are widely used during initial visits, as they are based on principles of learning theory, where children are guided through explanation and demonstration of procedures [14]. In contrast, the Tell-Play-Do technique adopts a more interactive approach by letting children to actively engage with dental tools in a simulated environment. This learning by doing method helps children better understand dental procedures and may enhance their sense of control and familiarity [15]. In recent years, the increasing use of smartphones among children has opened new avenues for behaviour management. Mobile-based dental applications provide an engaging platform that combines visual, auditory and interactive elements to explain dental procedures in a child-friendly manner. These applications often include animated simulations, step-by-step treatment representations and interactive features, which can help children, visualize the clinical process and reduce uncertainty [16]. As a result, smartphone-assisted modelling may serve as an effective modern alternative to conventional techniques by improving understanding and reducing anxiety prior to treatment. Hence, the present study evaluated the effectiveness of TSD, TPD and smartphone-assisted modelling in pediatric patients. In present investigation children aged 6-8 years were selected as this age category demonstrates adequate cognitive development to understand instructions and respond reliably to behaviour management techniques, while still being susceptible to dental anxiety. The RMS Pictorial Scale (RMS-PS) was used for anxiety assessment as it is a simple, reliable and child-friendly tool that enables young children to express their emotional state through visual representation, even with limited verbal communication skills [17]. Restorative treatment was chosen as the clinical procedure because it commonly involves anxiety-provoking stimuli such as sound, vibration and unfamiliar instruments, making it suitable for assessing the efficiency of different behaviour management strategies under standardized conditions. In the current research, all three behaviour management strategies showed a significant reduction in anxiety among children. The Tell-Show-Do technique was effective due to gradual familiarization, while Tell-Play-Do showed better results as it involved active participation and learning by doing. The greatest reduction in anxiety was observed with smartphone app-assisted modelling, which can be attributed to its interactive audiovisual approach that helps children better understand dental procedures and reduces fear. The better performance of the Tell-Play-Do technique can be attributed to its interactive nature, where children actively participate and become acquainted with dental instruments and procedures in a safe, non-threatening way. This learning by doing approach enhances understanding and promotes cooperative behaviour. These findings are consistent with previous studies. Vishwakarma et al. reported that Tell-Play-Do was more effective than live modelling in reducing anxiety and improving cooperation among children [18]. Similarly, the effectiveness of smartphone-based interventions observed in the present study is supported by Elicherla et al. who demonstrated that a mobile dental application significantly reduced dental anxiety compared to conventional techniques [19]. In addition, Rahaman et al. reported that the use of a dental treatment simulation app effectively reduced anxiety levels in pediatric patients by improving their familiarity with dental procedures [20]. The findings of the present study are consistent with previous literature emphasizing the effectiveness of interactive behaviour management techniques in pediatric dentistry. Khade et al. [21] reported that the Tell-Play-Do technique was more effective than the conventional Tell-Show-Do approach in reducing anxiety and improving cooperative behaviour among children. Similarly, Gaikwad et al. demonstrated that Tell-Play-Do and filmed modelling combined with Tell-Show-Do produced significantly better child behaviour compared to Tell-Show-Do alone. These findings suggest that play-based and modelling behavioural interventions enhance psychological preparation, reduce dental fear, and improve acceptance of dental procedures among pediatric patients [22]. The superior performance of smartphone app-assisted modelling in the present study may be explained by its ability to provide a combination of visual, auditory and interactive learning experiences. Such multimodal stimulation helps reduce fear of the unknown and enhances the child's understanding of the procedure, thereby improving acceptance of dental treatment [23, 24, 25]. Overall, the findings suggest that while conventional techniques remain effective, integrating interactive and technology-based approaches can further enhance behaviour management outcomes in pediatric dentistry. The study is constrained by a relatively small sample size, which may limit the generalizability of the findings. Furthermore, the use of a subjective pictorial scale may be influenced by individual interpretation.

## Conclusion:

We report that all behaviour management approaches effectively reduced dental anxiety in pediatric patients. Smartphone app-assisted modelling showed the greatest reduction in anxiety level, followed by Tell-Play-Do, while Tell-Show-Do was comparatively less effective. Incorporating interactive, technology-based methods may further enhance child cooperation during dental treatment.

## Figures and Tables

**Table 1:** Demographic distribution of study participants

**Variable**	**Group I (TSD)**	**Group II (TPD)**	**Group III (App)**	**p-value**
Age (years)	7.1 ± 0.8	7.0 ± 0.7	7.2 ± 0.6	>0.05
Male	6 (60%)	5 (50%)	6 (60%)	>0.05
Female	4 (40%)	5 (50%)	4 (40%)	>0.05

**Table 2:** Assessment of pre- and post-intervention RMS-PS scores within groups (Mean ± SD)

**Group**	**Preoperative**	**Postoperative**	**p-value**
TSD	3.8 ± 0.7	2.9 ± 0.6	<0.05*
TPD	3.9 ± 0.6	2.4 ± 0.5	<0.05*
Smart Phone App	4.0 ± 0.7	1.8 ± 0.4	<0.05*

**Table 3:** Post-hoc analysis (pairwise comparison between groups)

**Comparison**	**Mean Difference**	**p-value**
I vs II	0.6	<0.05*
I vs III	1.3	<0.05*
II vs III	0.7	<0.05*
*Statistically
significant (p < 0.05)
